# Anti-Inflammatory Role of Netrin-4 in Diabetic Retinopathy

**DOI:** 10.3390/ijms22094481

**Published:** 2021-04-25

**Authors:** Sergio Crespo-Garcia, Nadine Reichhart, Norbert Kociok, Sergej Skosyrski, Antonia M. Joussen

**Affiliations:** 1 Experimental Ophthalmology, Department of Ophthalmology, Charité—Universitätsmedizin Berlin, Freie Universität, Humboldt-University, The Berlin Institute of Health, Augustenburger Pl. 1, 13353 Berlin, Germany; nadine.reichhart@charite.de (N.R.); norbert.kociok@charite.de (N.K.); sergej.skosyrski@charite.de (S.S.); 2 Department of Biochemistry, Hospital Maisonneuve-Rosemont Research Center, University of Montreal, 5415 Blvd. Assomption, Montreal, QC H1T 2M4, Canada

**Keywords:** netrin-4, retina, diabetic retinopathy, chronic inflammation, in vivo

## Abstract

Diabetic retinopathy is characterized by dysfunction of the retinal vascular network, combined with a persistent low-grade inflammation that leads to vision-threatening complications. Netrin-4 (NTN4) is a laminin-related secreted protein and guidance cue molecule present in the vascular basal membrane and highly expressed in the retina. A number of studies inferred that the angiogenic abilities of NTN4 could contribute to stabilize vascular networks and modulate inflammation. Analyzing human specimens, we show that *NTN4* and netrin receptors are upregulated in the diabetic retina. We further evaluated a knock-out model for NTN4 undergoing experimental diabetes induced by streptozotocin. We investigated retina function and immune cells in vivo and demonstrated that NTN4 provides a protective milieu against inflammation in the diabetic retina and prevents cytokine production.

## 1. Introduction

Diabetic retinopathy (DR) is one of the most recurrent complications in diabetic patients and a driving cause of blindness in the working-age population [1]. During DR, blood vessel networks experience increased vascular permeability leading to tissue ischemia. If the ischemic burden is too big, patients develop proliferative diabetic retinopathy (PDR). Another consequence of vascular leakage occurs when extravasated fluid accumulates [2], leading to diabetic macular edema (DME). These vascular phenomena are typically vision-threatening and are accompanied by other entities such as microaneurysms, the thinning of the vessel basal membranes [3], and low-grade chronic inflammation [4].

Netrins are secreted laminin-related proteins that have been extensively described as key players in neuronal migration and axon guidance, but also in tumor formation, angiogenesis, and inflammation [5]. Netrin-4 (NTN4) is a member of the netrin family expressed by multiple cell types, including glial and endothelial cells, and is a constitutive member of the basal membrane of blood vessels [6,7,8]. Interestingly, NTN4 shares the laminin domain with netrin-1 (NTN1), and there is evidence that NTN4 could exert its angiogenic roles via indirect interaction with the NTN1 putative receptors Unc-5 netrin receptor B (UNC5B) and Deleted in colorectal carcinoma (DCC) [9]. The mounting evidence has demonstrated that the presence of NTN4 protects endothelial cells from inflammation and senescence [10]. Among multiple organs, NTN4 is highly expressed in the eye [7,10], and multiple studies suggested that its angiogenic properties might be crucial in disease-associated scenarios. For instance, it has been shown that NTN4 accelerates wound healing and decreases inflammation in the cornea [11]. On the contrary, the absence of NTN4 modulates pathological angiogenesis in the ischemic retina [6,9,12] and alters vascular architecture and immune cell turnover in the adult mouse retina [13]. However, these studies are limited to a few models and do not always reflect human pathology.

One of the most recurrent approaches to treat DR consist of targeting vascular endothelial growth factor (VEGF)-A. Targeting VEGF-A, however, is not always beneficial to the patients and is only effective at the late stages of disease when the damage cannot be repaired [14]. A recent work revisited the knowledge on NTN4 and other netrin receptors in DR and emphasized the number of basic research studies that could have a translational impact in using netrins as DR biomarkers [15]. However, little is known of its role in DR and/or if netrins can play a role at earlier stages of the pathology. In fact, there exists only one report showing that NTN4 is downregulated in the vitreous of patients with PDR, suggesting that NTN4 could exert an anti-angiogenic function [16].

In this study, interestingly, we report that mRNA expression of *NTN4* and other netrin receptors were found upregulated in the retina from human PDR patients. Furthermore, we demonstrate that despite not having an impact on visual function, NTN4 plays a protective role in inflammation in a DR proxy model, using a knock-out model expressing in vivo a reporter for mononuclear phagocytes.

## 2. Results

### 2.1. Netrin-4 Expression Increases in the Human Diabetic Retina

**Figure 1:**
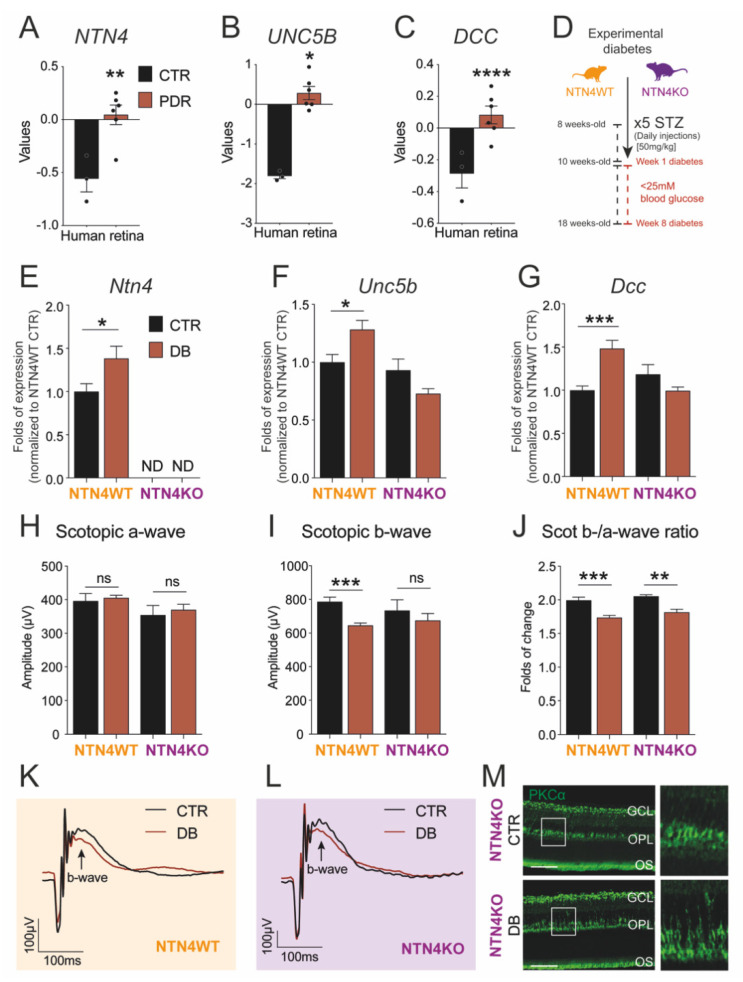
Diabetic retinopathy induces netrin-4 upregulation and impacts visual function. (**A**–**C**) Bar charts represent *NTN4* (**A**), *UNC5B* (**B**) and *DCC* (**C**) gene expression of human retina lysates comparing PDR patient samples (*n* = 6 specimen) to control (*n* = 3 specimen) Dataset: GSE60436. (**D**). Schematic illustrates the experimental diabetes model used via injection of streptozotocin (STZ) to obtain sustained hyperglycemia in both the NTN4WT and NTN4KO. (**E**–**G**). Bar charts represent mRNA folds of expression to NTN4WT control. Target gene expression of *Ntn4* (**E**), *Unc5b* (**F**), and *Dcc* (**G**) were normalized to *Ywhaz* expression (*n* = 3–8 animals per group and condition after 8 weeks diabetic). (**H**–**J**) Bar charts represent scotopic a-wave (**H**), scotopic b-wave (**I**), or the b-/a-wave ratio (**J**) of NTN4WT and NTN4KO animals 8 weeks after diabetes induction with their respective controls (*n* = 4–8 animals per group). (**K**,**L**). Scotopic ERG curve representing the average values for either NTN4WT (**K**) or NTN4KO (**L**) animals. (**M**) Representative micrographs with the expression of PKCα (green) in cross-sections from NTN4KO control and diabetic mice (*n* = 3 animals per group, 8 weeks of diabetes). White boxes indicate a detail of the micrograph (expanded in the right panel) depicting a slight delocalization of the bipolar cells in diabetes. Scale bar = 50 µm. The asterisk stands for significant *p*-values when compared to intra-strain control (* = *p* < 0.05; ** = *p* < 0.01; *** = *p* < 0.001; **** = *p* < 0.0001; ns = not significant using Student *t*-test). CTR, control. PDR, proliferative diabetic retinopathy. DB, diabetic.

Using publicly available transcriptomic data on human diabetic samples deposited at Gene Expression Omnibus (GEO), we demonstrated that transcript levels of *NTN4* were significantly upregulated in PDR (*n* = 6 specimen) when compared to age-matched control retinas (*n* = 3 specimen) (Figure 1A). Interestingly, this finding was correlated with the significant upregulation in the mRNA expression of the NTN1 receptors *UNC5B* (Figure 1B) and *DCC* (Figure 1C) as well.

### 2.2. Netrin-4 Expression Increases in Experimental Diabetic Retinopathy

To investigate the direct implication of NTN4 destabilizing the vascular network in a proxy model for DR, we experimentally induced DR in mice using streptozotocin (STZ) on mice lacking NTN4 expression (referred to as NTN4KO in this paper). Furthermore, mice were transgenically modified to harbor expression of the EGFP reporter in all myeloid cells to enable in vivo evaluation. Experimental diabetes was elicited via sustained hyperglycemia, and it was monitored throughout the experimental time-course. STZ-injected mice showed significantly high glucose levels starting at 2 weeks after STZ compared to citrate-injected controls for both strains (Table 1). We did not observe statistically significant differences in hyperglycemia among diabetic NTN4KO and its wildtype, referred to as NTN4WT, in this paper. These observations were correlated as well, with no statistically significant differences related to weight among strains (Table 2).

8 weeks after STZ injections, NTN4WT retina lysates showed a significantly higher mRNA expression of *Ntn4* compared to non-diabetic control. In the knock-out, *Ntn4* expression was undetectable (Figure 1E). The levels of expression of the NTN1 receptors *Unc5b* and *Dcc* were also significantly higher in the diabetic wildtype retina, but surprisingly not in NTN4KO mice (Figure 1F,G). These data contribute to previous evidence regarding the indirect modulation that NTN4 exerts on the receptors.

### 2.3. Netrin-4 Expression Does Not Alter Visual Function in Experimental Diabetic Retinopathy

Previously, we reported that adult NTN4KO shows a hampered inner retina function compared to wildtype [13]. Given that DR and, specifically, STZ-induced diabetes has a repercussion on visual acuity, we evaluated the function of retinal cell types by means of scotopic Ganzfeld electroretinography (ERG) in order to assess the impact of NTN4 in the diseased retina. After 8 weeks of diabetes, scotopic a-wave (related to photoreceptor performance) remained unaltered in all groups independently of the genotype (Figure 1H). Interestingly, the b-wave, related to bipolar cell and inner retina function, was significantly reduced in the diabetic NTN4WT with respect to the citrate-injected control, while in the NTN4KO showed only a trend albeit not significant (Figure 1I). Calculated b-/a-wave ratios confirmed that both NTN4WT and NTN4KO diabetic retinas had a reduced visual function compared to their respective strain controls, finding it more pronounced in the wildtype (Figure 1J–L).

Since we were able to identify that STZ leads to a reduced function of the inner retina independently on the genotype, we used the pre-synaptic and bipolar cell marker, PKCα, to validate inner retina integrity on sagittal sections. Evaluation of PKCα, however, indicated that bipolar cells were present in the diabetic retinas, and we could only observe a mild yet not significant alteration in the localization of the signal in the outer plexiform layer (OPL) (Figure 1M).

### 2.4. Lack of Netrin-4 Accentuates Low-Grade Inflammation in Diabetic Retinopathy

**Figure 2:**
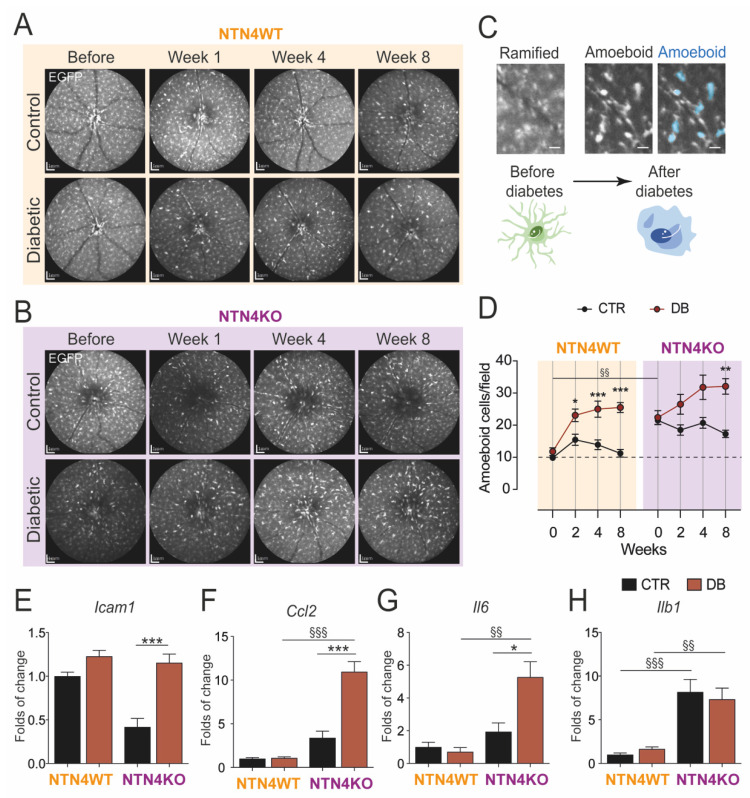
Netrin-4 modulates inflammation in the retina during diabetes. (**A**,**B**) Representative scanning laser ophthalmoscope images of one animal from either NTN4WT (**A**) or NTN4KO (**B**) during the time-course of diabetes (*n* = 10–13 animals per group) (**C**) Representative illustration of ramified mononuclear phagocytes (EGFP-positive) in the retina and their turnover to amoeboid after diabetes induction (indicated in blue in the right panel). Scale bar = 200 µm Schematic cells represent the turnover. (**D**) Quantification of amoeboid cells per field during the time-course of diabetes in the distinct studied groups. (**E**–**H**). Bar charts represent mRNA folds of expression to NTN4WT control. Target gene expression of *Icam1* (**E**), *Ccl2* (**F**), *Il6* (**G**), and *Il1b* (**H**) was normalized to *Ywhaz* expression (*n* = 4–8 animals per group). The asterisk stands for significant *p*-values when compared to intra-strain control (* = *p* < 0.05; ** = *p* < 0.01; *** = *p* < 0.001 using Student *t*-test) whereas § stands for significant *p*-values when comparison was made between mouse strains (§§ = *p* < 0.01; §§§ = *p* < 0.001 using 1-way ANOVA). CTR, control. DB, diabetic.

Given the increasing evidence that NTN4 could play a role in inflammation [10,11,13], we interrogated whether NTN4 participates in the low-grade chronic inflammation associated with DR. Our group has previously demonstrated the advantages of evaluating mononuclear phagocytes in vivo over the progression of retinal pathology. We, therefore, monitored monthly the mouse retina during the time-course of diabetes using a scanning laser ophthalmoscope (Figure 2A,B). Cell morphology was evaluated to determine the activated state of mononuclear phagocytes (EGFP-positive) in the retina. We discriminated ramified EGFP-positive cells from the activated amoeboid round-like (Figure 2C). Activated amoeboid-like cells were significantly in higher numbers in the NTN4KO retina at the baseline of the study. After diabetes induction, however, the number of amoeboid cells augmented rapidly in both genotypes following the same fashion until week 8 (Figure 2D). All and all, NTN4KO kept a consistently higher number of amoeboid mononuclear phagocytes during all the time-course independently of the treatment.

Gene expression levels of multiple typical inflammatory cytokines confirmed that, after 8 weeks of diabetes, the NTN4KO retinas had an overall higher mRNA expression of *Icam1* (Figure 2E), *Ccl2* (Figure 2F), and *Il6* (Figure 2G) compared to non-diabetic NTN4KO, and also when compared to their diabetic NTN4WT counterparts in the case of *Ccl2* (Figure 2F), *Il6* (Figure 2G) and *Il1b* (Figure 2H) expression. Taken together, these results indicate that in diabetes, NTN4KO has a more accentuated pro-inflammatory profile.

## 3. Discussion

Given the little knowledge existing on NTN4, our study evaluating DR features in vivo provides valuable insight into the understanding of netrin-4 in disease. The dual role of netrins has been a recurrent and controversial topic as many studies discussed that its function could be dose-dependent [9,17]. The use of a systemic NTN4 knock-out permits understanding the consequences of the lack of NTN4 in a pathologic scenario such as diabetes, with local damage in the retina.

Collectively, our findings suggest that NTN4 contributes to DR regulating the immune response associated with diabetes and favoring an anti-inflammatory milieu. Our meta-analysis on transcriptomic data from fibrovascular membranes from human patients demonstrated an increased expression of *NTN4* and provided novel evidence for the interaction of NTN4 and the NTN1 receptors UNC5B and DCC. Despite limited to PDR, these findings point to the relevance of NTN4 in human pathology. In addition, our model proved to be suitable to evaluate immune cells in the retina in vivo and demonstrated the rapid turnover of mononuclear phagocytes into an amoeboid state as early as 1 week after diabetes induction. Although NTN4KO presented generally higher basal levels of amoeboid mononuclear phagocytes, it maintained higher numbers of amoeboid cells throughout the time-course of diabetes. All and all, NTN4KO mice expressed higher mRNA levels of several cytokines, resulting in a more pro-inflammatory retina profile.

Upregulation of netrin receptors in the NTN4KO mice during diabetes supports the data on the indirect interaction ligand-receptor. The existing upregulation of NTN1 receptors in the absence of NTN4 in a DR animal model contributes to the line of thought that despite not having a direct interaction, NTN4 can modulate such receptors. Alternatively, a study on cerebral ischemia demonstrated that after stroke, NTN4 is upregulated in astrocytes and blood vessels of the ischemic core, and DCC but not UNC5B is upregulated in neuronal processes [18]. Another published study described *UNC5B* to be downregulated using human umbilical vascular endothelial cells in an angiogenesis model for gestational diabetes in the placenta [19]. We believe this discrepancy could be influenced, firstly, by the in vitro approach of the experiments, and that the tissue studied by Prieto and colleagues does not belong to the central nervous system and lacks the NTN4 neuronal and glial contribution [18,20]. To be precise, there is evidence that Müller glia not only express NTN4 but can also modulate angiogenesis after ischemic injury in the retina [20].

In view of the fact that diabetes is accompanied by low-grade inflammation, multiple studies identified activated microglia in DR in both human specimens and experimental murine models [21,22,23,24], as well as analyzed the impact that microglia activation has on the diabetic retina [22,23]. Cell signaling associated with inflammation involving multiple factors such as TNF, CCL2, IL-1β, IL-6, and IL-8 have been identified as important factors in DR, and most of them were present in the vitreous of patients suffering from DME or PDR [25,26,27]. A recent study by Zhang and colleagues demonstrated the anti-inflammatory properties that NTN4 exerts on the vascular endothelium and how NTN4 contributes to maintaining the homeostasis of the adult vasculature via modulating cellular quiescence [10]. In this same study, inhibition of NTN4 leads to an increase in monocyte adhesion and impaired endothelial barrier functions, supporting our in vivo data.

Even though the STZ model that we test in this study is a Type I diabetes model and does not resemble the proliferative features of PDR, it consistently reproduces other characteristics such as retina neuro-degeneration or vascular leakage, which are highly associated with the human disease. The advantage of the STZ as a DR proxy remains on its versatility since it can be advantageously induced in knock-out models. These, however, are important limitations to take into consideration, and the role of NTN4 should be explored in detail in additional diabetic models, including Type II diabetic ones, as well as in other translational approaches.

Altogether, this work constitutes a ground discovery to ongoing projects to explore mechanistically the role of NTN4 in disease and its contribution to the maintenance of vascular homeostasis through inflammatory modulation. The findings presented in this study pave the way to systematic translational approaches to unveil the role of NTN4 in degenerative and inflammatory-related retinal diseases such as DR and to evaluate the potential of NTN4 as a biomarker for the severity/progression of DR.

## 4. Materials and Methods

### 4.1. Curation of Human Retina RNA-Sequencing Data

Available gene expression omnibus (GEO) transcriptomic data on human diabetic samples were analyzed using the GEO2R tool online [28]. RNA-sequencing databases were obtained from fibrovascular membranes from proliferative diabetic retinopathy (PDR) patients as well as age-matched control retinas and are publicly available under the accession number GSE60436 at the time of this study [29].

### 4.2. Animal Models

We used the knock-out murine model previously described by our group lacking NTN4 expression but harboring the EGFP reporter in all myeloid cells (referred to as NTN4KO in this paper) [13]. The control strain used in this study was the MacGreen mice [30], which stands wildtype for NTN4 yet with the EGFP transgene in myeloid cells (referred to as NTN4WT in this paper).

All animals were housed in 12 h-12 h cycles of light-darkness with free access to water and chow. All animals enrolled in this study were males at the age of ~8 weeks.

### 4.3. Streptozotocin-Induced Experimental Diabetes

Diabetes was induced by repeated intraperitoneal injections of streptozotocin (STZ; 50 mg/kg) for 5 consecutive days. Control non-diabetic animals were injected with vehicle citrate buffer (pH = 4.6) in the same fashion. Weight and blood levels were monitored, and animals were considered diabetic only when blood glucose levels were ≥25 mM, and weight loss was not >20% of the initial baseline weight of the animal (Figure 1D). Animals were investigated in vivo over a period of 8 weeks after STZ injection.

### 4.4. Analysis of Mononuclear Phagocytes in the Retina In Vivo

Periodically before and after STZ administration, animals were subjected to pupil dilatation and were anesthetized with 100 mg/kg of weight ketamine and 12 mg/kg of weight xylazine. EGFP-expressing myeloid cells were tracked using the autofluorescence mode in a Spectralis HRA-OCT scanning laser ophthalmoscope with a 50° lens (Heidelberg Engineering, Heidelberg, Germany) as described previously [31]. During the imaging, the eyes were continuously moisturized to delay cataract formation.

Activation of mononuclear phagocytes was determined based on morphological assessment, and only cells with a round-like shape were considered (Figure 2C). Quantification was performed using a manual annotation in a masked fashion by an independent investigator. The number of amoeboid cells is expressed as per the field of imaging as obtained using the Spectralis HRA-OCT using the Heidelberg Eye Explorer software (Heidelberg Engineering).

### 4.5. Scotopic Electroretinography (ERG)

Animals were dark-adapted overnight. At the time of the experiment, pupils were dilated and mice anesthetized with 100 mg/kg of weight ketamine and 12 mg/kg of weight xylazine. Scotopic electroretinography (ERG) was investigated using a Ganzfeld bowl (Roland Consult, Brandenburg, Germany) according to previously published protocols [6]. Shortly, animals were subjected to increasing intensity flashes (from −4.0 log cd·s/m^2^ to 0.48 log cd·s/m^2^), and the signal was amplified by 10.000 with a bandpass filter from 1 to 300 Hz.

### 4.6. Immunolabeling of PKCα

Eye globes were enucleated, incubated in Davidson fixative overnight, and embedded in paraffin. Cross-sections of 5 µm were deparaffined and re-hydrated using baths with increased concentration of water. Tissue was pre-treated with citrate buffer (pH 6.0) and blocked with 5% BSA. PKCα was detected using the antibody cat# sc-208 (Santa Cruz, Newark, NJ, USA) overnight, followed by incubation with fluorochrome-conjugated species-appropriate secondary antibody. Incubation with isotype controls served as negative controls. Images were obtained using a Zeiss LSM 510 (Zeiss, Jena, Germany) with the software ZEN lite blue edition (Zeiss).

### 4.7. Quantitative PCR

After 8 weeks with sustained hyperglycemia, animals were sacrificed, and eyes enucleated and snap-frozen. Retina were rapidly dissected and mRNA extracted using RNAeasy mini kit (Quiagen, Hilden, Germany). cDNA was obtained using QuantiTect Reverse Transcription Kit (Quiagen, Hilden, Germany), and gene expression was evaluated using quantitative PCR using SYBR green PCR kit (Qiagen, Hilden, Germany) and analyzed using the ddCT method. Using the the geNorm tool described in (32) *Ywaz* was chosen as a housekeeping gene, and gene expression is charted as ratios and normalized to citrate control of the NTN4WT.

The primer sequences used in this study were: *Ntn4* (F: CCG CAGG CTTG AAT GGA GTA; R: TATCACACTTGGGCTGCCG); *Ntn1* (F: GGC TTG CAA AGCC TGT GAT; R: ACA GGA ATC TTG ATG CAA GGG); *Unc5b* (F: CTT CCA CCC TGT CAA CTT CA; R: ACA CAG GCC CGC GGT AGA T); *Dcc* (F: CAG CGG AGA CCC CAG AGA A; R:TAC TGT CCA CAC GCA CTG TT); *Icam1* (F: CCA CTG CCT TGG TAG AGG TG; R:GTC AGG ACC GGA GCT GAA AA); *Ccl2* (F: CTG GAG CAT CCA CGT GTT GG; R: TGA GCT TGG TGA CAA AAA CTA CAG C); *Il6* (F: CGT GGA AAT GAG AAA AGA GTT GTG; R: TCA TGT ACT CCA GGT AGC TAT GGT); *Il1b* (F: CTA ATA GGC TCA TCT GGG AT; R: GGA AGC AGC CCT TCA TCT TT); *Ywaz* (F: CAA AAA CAG CTT TCG ATG AAG CC; R: TTT CCC CTC CTT CTC CTG CT).

### 4.8. Statistical Analysis and Experimental Design

All experiments were performed at least 3 independent times. *n* stands for the biological replicates and is indicated for each experiment in the figure legends. Results are expressed as mean ± SEM unless indicated otherwise. We used the Student *t*-test when comparing two groups, 1-way ANOVA when comparing more than two groups at one time-point or 2-way ANOVA when comparing more than two groups in a time-course. ERG analysis was performed using the Kruskal Wallis test.

## Figures and Tables

**Table 1:** Blood glucose levels after diabetes induction ^1^.

Weeks after STZ		Week 0		Week 2		Week 4		Week 8	
NTN4WT	Control	15.1 ± 0.9		14.7 ± 0.8		15.8 ± 0.4		17.4 ± 0.6	
	Diabetic	18.1 ± 0.3	ns	32.1 ± 0.8	***	32.7 ± 0.7	***	39.0 ± 0.6	***
NTN4KO	Control	16.6 ± 0.9		15.2 ± 0.5		14.9 ± 0.7		17.8 ± 1.8	
	Diabetic	17.2 ± 0.4	ns	31.6 ± 0.8	**	35.5 ± 1.9	***	36.8 ± 0.8	***

^1^ All data are expressed in mM as mean ± SEM. The asterisk stands for significant *p*-values when compared to intra-strain control (** = *p* < 0.01; *** = *p* < 0.001; ns = not significant using 2-way ANOVA).

**Table 2:** Weight after diabetes induction ^1^.

Weeks after STZ		Week 0	Week 2	Week 4	Week 8
NTN4WT	Control	23.8 ± 0.0	23.8 ± 0.0	26.0 ± 0.0	28.2 ± 0.0
	Diabetic	24.8 ± 0.0	24.5 ± 0.0	24.9 ± 0.0	25.8 ± 0.0
NTN4KO	Control	24.6 ± 0.0	25.6 ± 0.0	27.2 ± 0.1	29.2 ± 0.1
	Diabetic	24.6 ± 0.0	23.8 ± 0.0	24.4 ± 0.0	25.1 ± 0.0

^1^ All data are expressed in grams (g) as mean ± SEM.

## Data Availability

All data produced in this study are available in this manuscript. If further information is required, please contact the corresponding authors, S.C.-G. (sergiocrespogarcia@gmail.com) or A.M.J. (antonia.joussen@charite.de), upon reasonable request.
